# Digital light processing 3D printing for microfluidic chips with enhanced resolution via dosing- and zoning-controlled vat photopolymerization

**DOI:** 10.1038/s41378-023-00542-y

**Published:** 2023-08-15

**Authors:** Zhiming Luo, Haoyue Zhang, Runze Chen, Hanting Li, Fang Cheng, Lijun Zhang, Jia Liu, Tiantian Kong, Yang Zhang, Huanan Wang

**Affiliations:** 1 School of Biomedical Engineering, Shenzhen University Health Science Center, Shenzhen, 518000 P. R. China; 2 State Key Laboratory of Fine Chemicals, Frontiers Science Center for Smart Materials Oriented Chemical Engineering, School of Bioengineering, Dalian University of Technology, Dalian, 116024 P. R. China; 3 Third People’s Hospital of Dalian, Dalian Eye Hospital, Dalian, 116024 P. R. China; 4 Central Laboratory, The Second Affiliated Hospital of The, Chinese University of Hong Kong, Shenzhen, 518172 P. R. China

**Keywords:** Engineering, Materials science

## Abstract

Conventional manufacturing techniques to fabricate microfluidic chips, such as soft lithography and hot embossing process, have limitations that include difficulty in preparing multiple-layered structures, cost- and labor-consuming fabrication process, and low productivity. Digital light processing (DLP) technology has recently emerged as a cost-efficient microfabrication approach for the 3D printing of microfluidic chips; however, the fabrication resolution for microchannels is still limited to sub-100 microns at best. Here, we developed an innovative DLP printing strategy for high resolution and scalable microchannel fabrication by dosing- and zoning-controlled vat photopolymerization (DZC-VPP). Specifically, we proposed a modified mathematical model to precisely predict the accumulated UV irradiance for resin photopolymerization, thereby providing guidance for the fabrication of microchannels with enhanced resolution. By fine-tuning the printing parameters, including optical irradiance, exposure time, projection region, and step distance, we can precisely tailor the penetration irradiance stemming from the photopolymerization of the neighboring resin layers, thereby preventing channel blockage due to UV overexposure or compromised bonding stability owing to insufficient resin curing. Remarkably, this strategy can allow the preparation of microchannels with cross-sectional dimensions of 20 μm × 20 μm using a commercial printer with a pixel size of 10 μm × 10 μm; this is significantly higher resolution than previous reports. In addition, this method can enable the scalable and biocompatible fabrication of microfluidic drop-maker units that can be used for cell encapsulation. In general, the current DZC-VPP method can enable major advances in precise and scalable microchannel fabrication and represents a significant step forward for widespread applications of microfluidics-based techniques in biomedical fields.

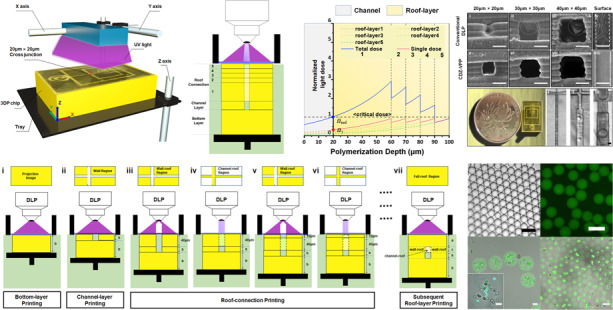

## Introduction

Microfluidic chips have been widely used as a powerful tool for miniaturization applications in three-dimensional (3D) cell culture^1–3^, drug screening and testing^4–6^, organ-on-a-chip^7–9^, microencapsulation^10–12^, and materials microfabrication^13–15^. Conventional technologies for the fabrication of microfluidic chips, including soft lithography and hot capillary fabrication process, have shown limitations such as complicated fabrication process, low productivity, and high cost^16,17^. Specifically, for the generation of complex 3D microstructures, these techniques typically use two-dimensional templates to establish the structure in a layer-by-layer manner, which requires precise alignment and the introduction of chemical bonding to fabricate a mechanically stable, multiple-layered chip^11,18^. These strategies are cost- and labor-intensive and require well-trained professional skills for chip fabrication. In addition, these techniques typically allow the fabrication of only one chip in each batch, resulting in low productivity and uncontrollable batch differences^17^. Moreover, it remains a challenge to achieve bonding chemistry between layers that can enable long-term mechanical and structural integrity to tolerate the high internal pressure upon inputting highly viscous liquids^19–21^. All these drawbacks limit the widespread applications of microfluidic-based devices in biomedical applications, and the development of new microfabrication strategies for microfluidic chips with improved cost-efficiency and scalability is essential.

**Fig. 1:**
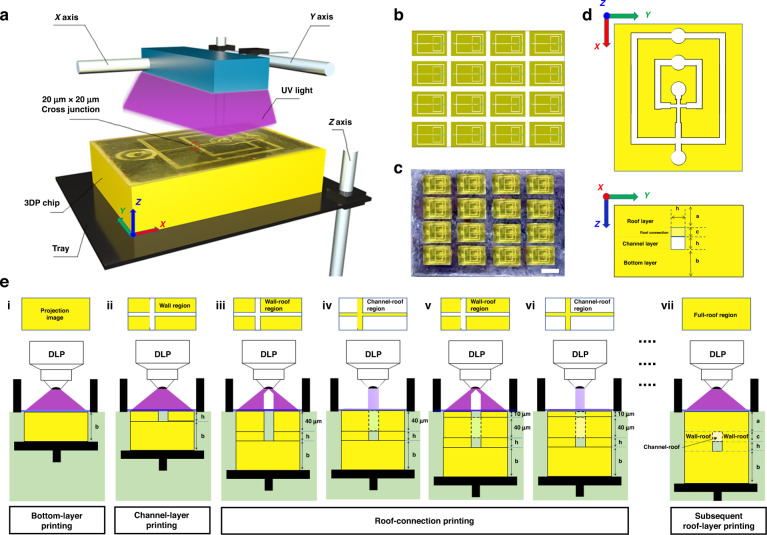
Scheme showing the design principle of the DZC-VPP fabrication process. **a** Schematic image showing the setup of the DZC-VPP apparatus. **b** CAD design of batch printing. **c** Photograph of batch printing productions. The scale bar is 1 cm. **d** Orthographic views of the channel structure (x-y axis) and the drop-maker microfluidic chip design with the bottom layer (height: b μm), channel layer (height: h μm, width: h μm), connection layer (height: c μm), and roof layer (height: a μm). **e** The process design of DZC-VPP. Fabrication of the bottom layers (**i**) and channel layers (**ii**) using the conventional equal-step DLP process. **iii**. Fabrication of the first wall-roof connection layer, in which the UV dose is equal to the bottom layer; **iv**. Fabrication of the first channel-roof connection layer using a critical dose; **v**. Fabrication of the subsequent wall-roof connection layer; **vi**. Fabrication of the subsequent channel-roof connection layer; **vii**. Fabrication of the roof layers using the conventional DLP process

3D printing techniques have recently attracted increasing attention as they allow the innovative design and customized manufacture of complicated structures with micrometer-scale resolution^22,23^. Common 3D printing technologies have been able to achieve millimeter-scale structure manufacturing based on typical extrusion-based printing process^24^. Based on a multi-material printing process, sub-millimeter-scale structures can be fabricated with the introduction of sacrificial support materials by melt-electro writing^25^ and fused deposition modeling technologies^26,27^. These methods have already met the scale requirement of biological analysis or chemical synthesis, but still difficult for microfluidic device fabrication for single-cell encapsulation and analysis^17^. Particularly, DLP printing based on layer-by-layer vat photopolymerization has been increasingly used for microfabrication because of its high resolution up to tens of microns, rapid processing speed, ease of operation, and versatility in resin materials^22,23,28^ (Fig. 1a). These advantages make the DLP technique favorable for the fabrication of microfluidic chips, which are expected to possess the desired resolution, optical transparency, smooth surfaces inside the channels, and sufficient mechanical stability to tolerate high pressure^19^. Although conventional DLP processing strategies have led to a significant breakthrough in preparing microchannels, the cross-sectional dimensions of the obtained channels can only reach several hundreds of microns at best or can achieve sub-100-micron resolution but only in a small printing area^29–32^, which is still insufficient for various applications requiring most channels to be smaller, such as single-cell manipulation and encapsulation (typically <50 μm)^12,33,34^. Since these DLP processes invariably utilize an equal-step printing strategy, irradiance overdosing is almost unavoidable, as UV irradiation stemming from the neighboring layers can accumulate due to the transparency of the resin; this normally leads to channel blockage since the penetration of the additional light can induce polymerization of the resin residing in the channels^35^. In contrast, if insufficient irradiance is applied, the polymerization of the resin can be incomplete, resulting in poor mechanical stability between stacked layers and potential cytotoxicity due to the leakage of photoinitiators and unreacted monomers^36,37^. These difficulties in precise control of the UV irradiance accumulated during layer-by-layer resin photopolymerization can significantly compromise the printing fidelity of DLP fabrication, which becomes even more pronounced upon the fabrication of small channels with dimensions of tens of microns.

To this end, recent studies have proposed novel strategies combined with mathematical models to carefully control resin polymerization and specific parameter control to guide the design and DLP fabrication of microfluidic devices. These approaches made it possible to quantify the polymerization parameters of resins with different formulations, such as optical irradiance and UV wavelengths^38–40^. However, due to the transparency of the solidified resin, UV irradiance can still penetrate into the internal cavity of the channel space, resulting in overcuring resin polymerization and channel blockage. Thus, the minimum printed structures of these methods can reach sub-100 microns in a small area at best^32,35,39,40^, which is not suitable for many delicate applications such as single-cell encapsulation and analysis. Alternatively, novel strategies for DLP processing via optimization of the mathematical model combined with customized equipment and materials were proposed to improve the printing resolution of microchannel structures^35,40^. The finest channel structure they obtained can reach 20 μm (approximately three times the pixel length) using a specially designed 3D printer equipped with a specific combination of a high-resolution light engine, a specific UV absorber, and a specialized UV light source^40^. The use of the UV absorber may introduce cytotoxicity regarding cell-related applications, which also limits its widespread application^41^. Moreover, by exposing multiple overlapping images to a single layer to control the dose on a pixel-by-pixel basis with individual exposure times, a 15 μm × 15 μm partial structure (2 × 2 pixels) can be fabricated within one printing layer^32^; however, this resolution still cannot meet the wide-area precision machining requirements of delicate applications. Therefore, improving the DLP resolution of microchannels remains a challenge, and developing a novel DLP strategy to achieve the desired fidelity and mechanical stability of chips is imperative.

We herein developed a new DLP printing strategy for the high-resolution and scale-up fabrication of microfluidic devices by dosing- and zoning-controlled vat photopolymerization (DZC-VPP). Simply using a commercial DLP printer (lateral optical resolution 10 µm, MicroArch 140 S, BMF Material Technology Inc) and resin (HTL, BMF Material Technology Inc), DZC-VPP can significantly improve the fabrication resolution of a long channel’s cross-section to 20 μm × 20 μm, which is only two times longer than the lateral optical resolution of this commercial DLP printer (10 μm × 10 μm per pixel). Specifically, for printing the microchannels, we proposed a mathematical model to precisely predict the accumulated UV irradiance for each layer of resin photopolymerization and to guide the fabrication of complex microstructures. By fine-tuning the printing parameters, including optical irradiance, exposure time and projection region, and step distance, we can precisely tailor the penetration irradiance stemming from the photopolymerization of the neighboring resin layers, therefore avoiding channel blockage due to UV overexposure or compromised bonding stability owing to insufficient resin curing. Remarkably, in comparison with the conventional fabrication process of polydimethylsiloxane (PDMS) or glass capillary microfluidic devices, this strategy can enable the one-batch fabrication of up to 16 drop-maker microfluidic chips with channel dimensions of 20 μm × 20 μm using only a commercial DLP printer and resin (Fig. 1b, c). We also showed that these fabricated chips can be further used for cell encapsulation in droplets/microgels, indicating the biocompatibility of this fabrication strategy. In general, the current DZC-VPP technique can enable major advances in precise and scalable microchannel fabrication and represents a significant step forward for the widespread application of microfluidics-based devices in biomedical applications.

## Results and discussion

### Mathematical model to predict the characteristic parameters of the resin

**Fig. 2:**
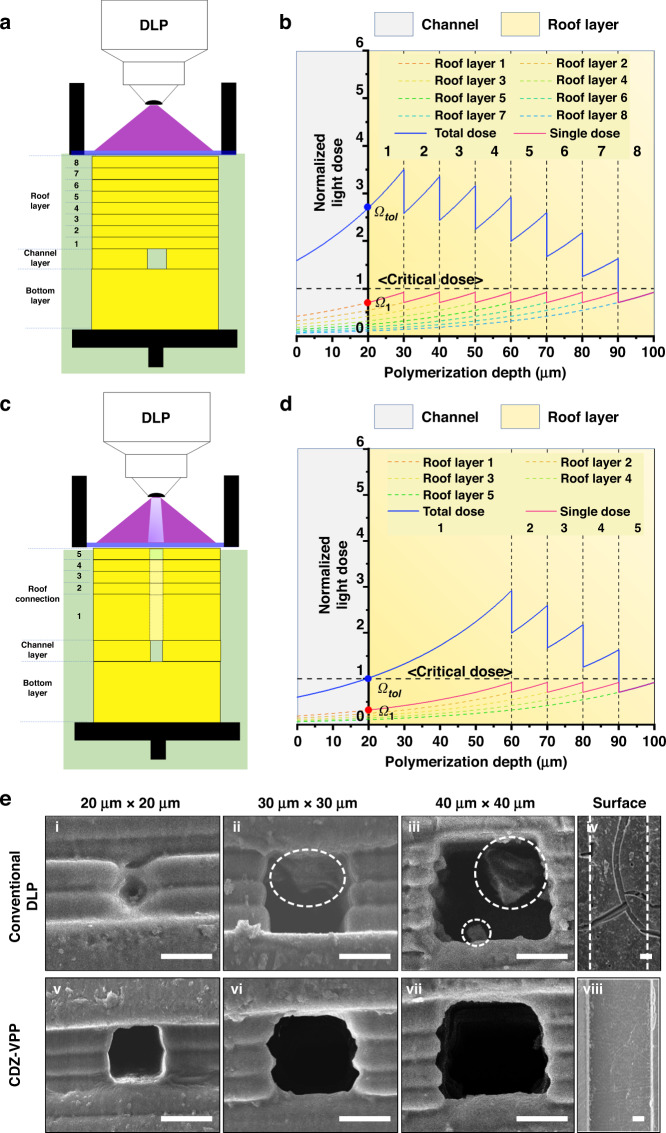
High-resolution microchannel fabrication by the DZC-VPP strategy in comparison with conventional DLP processing. **a**, **c** Schematic illustrations showing microchannel fabrication by the DZC-VPP strategy (**c**) in comparison with conventional DLP (**a**). **b**, **d** The distribution of UV irradiance of each projection along the vertical direction that accumulated during the fabrication of the channel layers and roof layers using the DZC-VPP strategy (**b**) in comparison with the conventional DLP (**d**). **e** Scanning electron microscopy (SEM) images of the cross-section at the edge of devices and the inside top surfaces (width 100 μm) of the fabricated microchannels. White dotted circles show the residual polymerized resin within the channel space. White dotted lines show the channel region covered by residual polymerized resin. Scale bars are 20 μm

The key to the DLP printing of ultrasmall microchannels (below 50 microns) is to precisely control the dosing of UV irradiation just slightly higher than the critical dose that is needed to cure the resin, especially for the fabrication of the roof layer^35^. Conventional equal-step DLP strategies typically apply stepwise UV irradiation with equal penetration depth to polymerize the resin in a layer-by-layer manner (Fig. 2a). For each individual layer of polymerized resin, the UV irradiation applied to the resin can be estimated using the following mathematical model (Eq. 1)^35,40^.

1 $${L}={{h}}_{{a}}{ln}\frac{{{It}}^{*}}{{{D}}_{{c}}}$$

where *I* represent the intensity of the UV irradiance, *D*_c_ is the critical irradiation dose required for the polymerization of a certain type of resin, and ${h}_{a}$ is the characteristic penetration depth of the resin. Upon UV irradiation for an exposure time of t^*^, a resin solution with a depth of *L* can be polymerized. Apparently, *D*_*c*_ and *h*_*a*_ are characteristic variables for different resins, which can be quantitatively characterized by experiments. Specifically, we tested the validity of this theoretical model using a commercial resin (HTL, BMF Material Technology Inc., Shenzhen, China). By varying the exposure time *t* (0.8–1.2 s) of the UV light with the optical irradiance *I* ranging from 10 to 30 mW/cm^2^, solidified samples with different polymerization depths *L* ranging from 10 to 45 μm can be achieved (Fig. S1, Table S1, Supporting Information). These results allowed us to quantify the characteristic parameters *h*_*a*_ and *D*_*c*_ of the commercial resin.

After determining *h*_*a*_ and *D*_*c*_, the UV irradiation dose received by the resin at a depth of *z* in the vat can be calculated using the following mathematical model (Eq. 2)^35^.

2 $$\Omega \left({z},{t}\right)=\frac{{t}^{*} {I}}{{{D}}_{{c}}}{{e}}^{-\frac{{z}}{{{h}}_{{a}}}}$$

where Ω is the received irradiation dose normalized by the critical dose *D*_*c*_^35^. Therefore, Ω = 1 indicates a specific irradiation dose received by the resin to ensure appropriate polymerization, while Ω < 1 or Ω > 1 suggests incomplete or overdosing photopolymerization of the resin, respectively. For microchannel fabrication using the conventional equal-step DLP strategy (Fig. 2a), the residual resin in the channel space can receive an overdose of UV irradiation penetrating the previously printed steps, resulting in resin overcuring and channel blockage. Thus, predicting the dosing of penetrated UV irradiance is critical to enable precise control of resin polymerization and printing resolution. The accumulation dose of UV irradiance for each individual layer of resin can be calculated by Eqs. 3, 4^35^.

3 $${\Omega}_{{n}}\left({z},{t}\right)=\frac{{t}^{*} {I}}{{{D}}_{{c}}}{{e}}^{-\frac{{{z}}_{{l}}}{{{h}}_{{a}}}}$$

4 $${\Omega}_{{tol}}\left({z},{t}\right)=\mathop{\sum}\limits_{{n}=1}^{{N}}{\Omega}_{{n}}\left({z},{t}\right)$$

where *n* ∈ [1, N], and *N* represents the total number of projection steps preceding the current step of resin photopolymerization. Ω_*n*_ is the penetrated UV irradiance generated from the UV exposure of the *N*^th^ step that will be accumulated at the first step of the polymerized resin, and *z*_*l*_ is the distance between the first and the *N*th step. For instance, as shown in Fig. 2b, ${\Omega }_{5}$ is the penetrated UV irradiance generated during the fifth projection step that reached the first step of polymerized resin. Ω_tol_ represents the total accumulation of UV irradiance generated by the previous *n* steps of UV exposure that are collectively received by the first step. Thus, for the fabrication of a microchannel structure using the conventional equal-step DLP strategy, the overall UV irradiance can accumulate to reach almost three times higher than the critical dosing necessary for resin polymerization (Fig. 2b); this will cause overcuring of the residual resin in the channel space and thereby block the channel. Therefore, to address this problematic issue and enhance printing accuracy, fine-tuning the accumulation of UV irradiance to less than the critical dose (i.e., Ω_tol_ ≤ 1) is the key. In general, this mathematical model enabled us to involve not only the parameter prediction of the commercial 3D printer (including irradiation parameters and mechanical accuracy) but also the parameterization of the resin materials (including *D*_*c*_ and *h*_*a*_) and to precisely predict and control the overall UV accumulation at different positions of the printed constructs, which is essential for the fabrication of microchannels with smaller dimensions. Therefore, our model offered a more comprehensive way to calculate the threshold of resin polymerization.

### Design rationale and experimental setup for microchannel fabrication using the DZC-VPP strategy

Based on this understanding, we herein proposed a modified DLP printing strategy for the fabrication of substantially small microchannels via dosing- and zoning-controlled vat photopolymerization (DZC-VPP). Unlike conventional DLP strategies that use the same printing parameters for each equal-depth layer, the DZC-VPP strategy divides the microchannel into the bottom layer, channel layer, and roof layer (Figs. 1d, 2c). Specifically, the settings for roof-layer printing were completely different compared to the other layers (Fig. 1e). To illustrate the DZC-VPP processing method, we used a classical cross junction-shaped drop-maker microchannel with a channel dimension of 20 μm × 20 μm as a model and described the process as follows (Fig. 1d).

(1) The bottom layer with a thickness of 1500 μm was printed using a conventional DLP process (Fig. 1e (i)) to provide a solid and smooth bottom surface for the microchannel. The following parameters were used: UV irradiance *I* = 30 mW/cm^2^, exposure time *t* = 0.8 s, and step distance *z* = 20 μm.

(2) The channel layer with a height of 20 μm was printed using the same parameters as (1) except that the step distance *z* was set at 10 μm (Fig. 1e (ii)). The corresponding projection image of the cross-junction channel is shown in Fig. 1d.

(3) Regarding the fabrication of the roof layer, we proposed to separately print wall-roof connections (Fig. 1e (iii, v)) and channel-roof connections (Fig. 1e (iv, vi)). Therefore, the zoning of resin polymerization for each projection step needs to be separately carried out, and different UV doses can be applied. Specifically, the above-mentioned mathematical model was used to predict UV accumulation after step-wide printing. To prevent overdosing or insufficient resin polymerization, we set the step distance for printing the first step of the roof layer to 40 μm, and the following part was printed with a step distance of 10 μm (Fig. 1e (iii–vii), **2C**).

(a) For the wall-roof connection, as this structure is designed to provide mechanical support to the chip, overcuring of the resin can be accepted. Thus, a higher UV irradiance and longer exposure time were used (Fig. 1e (iii, v)); UV irradiance *I* = 30 mW/cm^2^, exposure time *t* = 1.35 s. According to Eqs. 3 and 4, the penetrated UV irradiation eventually accumulated at the interface of the wall-roof connection will be 1.26 (z = 40 μm, Fig. 1e (iii)), which is enough for the complete curing of this layer. Besides, a short step distance (z = 10 μm) was used to ensure complete polymerization and sufficient bonding stability between steps (Fig. 1e (v)).

(b) For the channel-roof connection, the irradiation dose should be precisely controlled to reduce excessive UV accumulation. With the aid of our mathematical model (Part 2.1, Eqs. 3 and 4), the exposure energy t* *I* needs to be lower than 5.4 mJ/cm^2^. Although any combination of exposure time t* and irradiance intensity *I* can be applicable as long as the overexposure energy is ≤5.4 mJ/cm^2^ (*I* = 20 mW/cm^2^, t* = 0.25 s, for example), an irradiation time that is too short is difficult to control precisely in experiments. Therefore, we herein set the printing parameters as optical irradiance *I* = 10 mW/cm^2^ and exposure time *t* = 0.5 s. As such, the overall accumulation of UV exposure at the interface of the channel-roof connection ${\Omega }_{{{\mathrm{tol}}}}$ will be 0.92 (Fig. 2d), which can avoid the overcuring of the residual resins in the channel space.

(4) For printing structures far away from the channel area, the same printing parameters as the bottom layer can be applied since the accumulation of UV penetration can be ignored (Fig. 1e (vii)).

In comparison to conventional printing strategies, this printing path precisely divided the processing of microchannel printing into the bottom layer, channel layer, and roof layer, wherein the printing of the roof layer was further divided into wall-roof connections (Fig. 1e (iii, v)) and channel-roof connections (Fig. 1e (iv, vi)). Such zoning control of resin polymerization for each projection step can enable more precise control over local resin polymerization and prevent overdosing polymerization of the channel region. Consequently, we successfully printed channels with significantly higher resolution, reaching up to twice the size of the printing pixel of the printer.

### Printing fidelity of the DZC-VPP strategy for microchannel fabrication

We further evaluated the printing quality of the DZC-VPP approach compared with the conventional DLP (see details in Materials and methods). Microchannels with a cross-section from 20 μm × 20 μm to 40 μm × 40 μm were printed by the DZC-VPP method and compared with those fabricated by conventional DLP. SEM observations showed that conventional DLP led to severe channel blockage and poor fidelity for channels with different dimensions due to the accumulation of excessive UV exposure (Fig. 2ei-iv). Especially for the 20 μm × 20 μm channel, there was hardly any channel structure that could be formed using conventional DLP (Fig. 2ei). Besides, the residual polymerized resin in the channel space cannot be easily removed (Fig. 2eii, iii), thus a rather rough surface would appear inside the channels (Fig. 2eiv), which can be attributed to the overcuring of resin due to excessive UV accumulation. In contrast, the DZC-VPP processing resulted in the formation of microchannels with significantly improved printing fidelity compared to those fabricated by the conventional DLP strategy (Fig. 2ev–viii). For the fabrication of 20 μm × 20 μm channels, we observed a square-shaped cross-section of the channel structure generated by the DZC-VPP strategy (Fig. 2ev). Owing to the meticulous regulation of UV irradiance at each layer during the fabrication process, the elimination of residual unpolymerized resin located on the top surface of the channel becomes relatively easier. Consequently, this technique enables the creation of remarkably smoother internal surfaces within the microchannels (Fig. 2eviii), which is essential for fluid manipulation using microfluidic devices since smoother surfaces cannot significantly influence the fluid state at the microscopic scale.

Another advantage of our DZC-VPP 3DP method for microchannel fabrication is its high scalability. Herein, we used a commercial DLP printer with a working platform of 10 cm × 5 cm, which allowed one-batch production of at least 16 drop-maker microfluidic chips (1 cm × 2 cm each) with high fabrication resolution in 6 h (0.375 h per chip, Fig. 1b, c and S3, Supporting Information), and the average cost for each chip can be reduced to $0.24. This is much lower than the material ($1.03) and man-hours (2 h per chip) costs of chips with similar structures produced by conventional soft-lithography methods^12,33^.

### Mechanical stability of the fabricated constructs using the DZC-VPP strategy

**Fig. 3:**
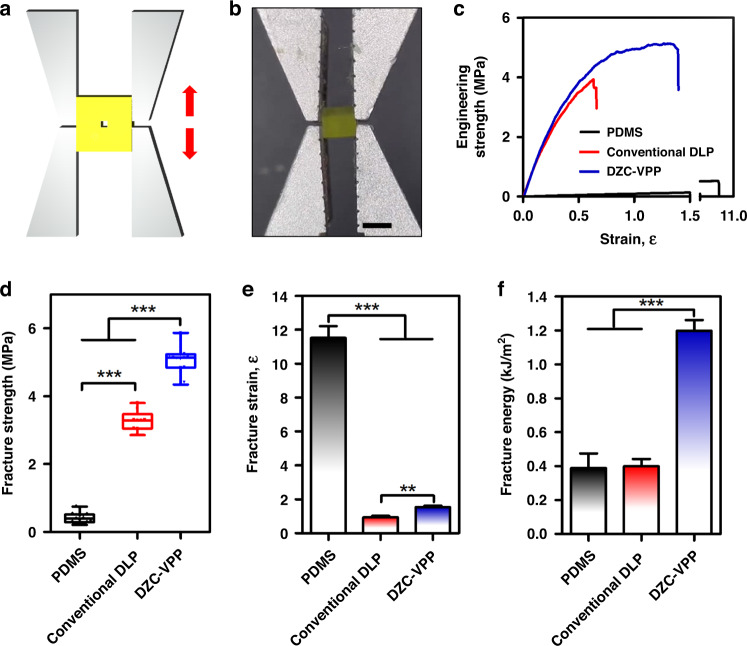
Mechanical stability tests of the fabricated microfluidic chips. **a**, **b** Schematic illustration (**a**) and photograph (**b**) showing the tensile tests to evaluate the bonding stability of the cubic samples (thickness of 200 μm, containing a 100 μm × 100 μm channel) fabricated by the DZC-VPP strategy in comparison to conventional DLP processing. The scale bar is 5 mm. **c** Stress‒strain curves of the fabricated resin samples based on different chip fabrication processes. **d**–**f** Fracture strength (**d**), fracture strain (**e**), and fracture energy (**f**) of PDMS, conventional DLP, and DZC-VPP chips. (**p < 0.01, ***p < 0.001)

We further evaluated the mechanical stability of the printed microfluidic devices fabricated using the DZC-VPP strategy in comparison with conventional DLP processing; this is one of the most important features for the applications of microfluidic chips since devices for high-throughput generation^18,42^ are required to tolerate high liquid pressure. To this end, tensile tests were applied to evaluate the binding stability between layers after the printing process (Fig. 3a, b). Samples fabricated by the conventional DLP process resulted in a fracture stress of 3.28 ± 0.15 MPa (Fig. 3c, d, conventional DLP), which was almost one magnitude higher than that of the PDMS chips fabricated by the soft lithography technique (0.41 ± 0.08 MPa, Fig. 3c, d, PDMS). As conventional DLP strategies typically print the channel and roof layers without delicate design, the irradiation doses of the channel layer and roof layer have to be precisely controlled to avoid excessive curing of the resin in the microchannel region; this, however, results in suboptimal binding strength between layers due to inadequate resin polymerization. Thus, although the stress‒strain curve of the DZC-VPP chip almost overlapped with that of the conventional DLP chip in the low-strain region (ε < 0.5, Fig. 3c), the DZC-VPP chip displayed significantly higher fracture stress (5.07 ± 0.20 MPa) and strain (ε = 1.53 ± 0.09) than that of the conventional DLP chip (Fig. 3c, d, DZC-VPP). In comparison, PDMS chips exhibited enhanced capability to tolerate larger deformation due to the high elasticity of silicone (fracture strain ε = 11.52 ± 0.63, Fig. 3e). We further quantified the fracture energy and noticed that DZC-VPP chips exhibited almost three times higher fracture energy (1.20 ± 0.08 kJ/m^2^) than conventional PDMS (0.39 ± 0.07 kJ/m^2^) and DLP chips (0.40 ± 0.03 kJ/m^2^, Fig. 3f). These findings suggested that the DZC-VPP strategy can improve not only the printing resolution but also the mechanical stability of the fabricated microfluidic chips.

### Generation of droplets and microgels using the fabricated microfluidic devices

**Fig. 4:**
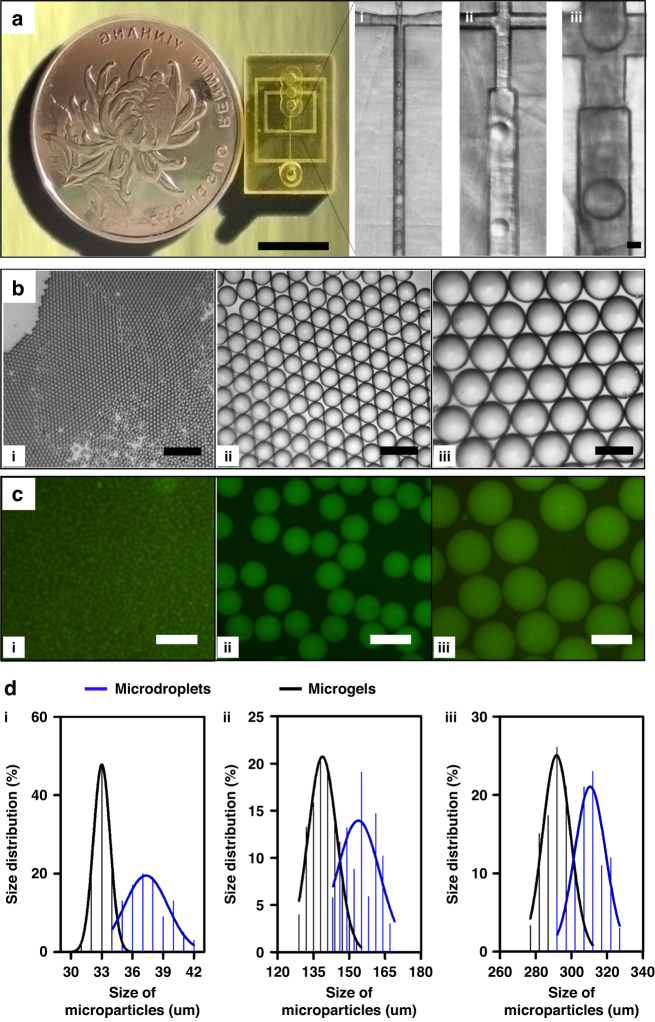
Experimental data of DZC-VPP chips for the customized production of microdroplets and microgels. **a** Photograph shows the volume of a single DZC-VPP chip in comparison to the coin, and the microscope images show flow patterns generated in the microfluidic device at different channel sizes of 20 μm (i), 100 μm (ii), and 300 μm (iii). Scale bars are 1 cm in photographs and 100 μm in microscope images. **b** Representative microscope images of microdroplets generated by DZC-VPP chips with channel sizes of 20 μm (i), 100 μm (ii), and 300 μm (iii). Scale bars are 300 μm. **c** Representative confocal microscopic images of microgels generated by DZC-VPP chips with channel sizes of 20 μm (i), 100 μm (ii), and 300 μm (iii). Scale bars are 300 μm. **d** Size distribution of microdroplets and microgels generated by DZC-VPP chips (five chips for each sample) with channel sizes of 20 μm (i), 100 μm (ii), and 300 μm (iii)

We further performed experiments to demonstrate the functionality and repeatability of the fabricated microfluidic devices, wherein classical cross-junction drop-maker devices were prepared and utilized for droplet-based microfluidic applications. Specifically, we tested the printed microchannels for droplet and microgel fabrication, wherein chips with cross-section dimensions of 20 μm × 20 μm, 100 μm × 100 μm, and 300 μm × 300 μm were printed (Fig. 4a). Monodisperse droplets can be fabricated in a continuous manner using these chips operating in the dripping regime (Fig. 4a (i–iii)). For the microfluidic generation of droplets, pure water was used as the aqueous phase and fluorocarbon oil (HFE7100) with 1 w/v% Krytox-polyethylene glycol (PEG)-Krytox surfactant as the continuous phase to prepare monodisperse aqueous droplets (Fig. 4b). The size of the fabricated droplets can be controlled by the dimension of the microchannels, with the size of the resulting droplets increasing with the channel size of the chips (Fig. 4d (i–iii)). Specifically, for the smallest channel with a cross-section of 20 μm × 20 μm, the average droplet size from multiple chips was as small as 37.65 ± 1.86 μm with a coefficient of variation (CV) <5% (Fig. 4d).

We further prepared microgels using microfluidic chips based on a previously reported alginate system^18^. Specifically, we introduced 1% alginate and 50 mM Ca-EDTA as the aqueous phase and 10 v/v% 1H,1H,2H,2H-perfluoro-1-octanol in combination with HFE7100 as the continuous phase with 0.1 v/v% acetic acid as the crosslinking initiator. After the aqueous droplets were formed at the first cross-junction, the acetic acid in the oil phase can diffuse into the droplets and trigger calcium release to crosslink alginate, and form monodispersed microgels (Fig. 4c). Consequently, size-controllable and highly monodisperse microgels can be produced, wherein the size of the microgels can also be fine-tuned by the dimension of the printed chips. For instance, microgels with an average size of 34.12 ± 1.74 μm can be prepared using multiple chips with 20 μm × 20 μm cross-junction (Fig. 4di). These results demonstrated that our DZC-VPP strategy could allow the fabrication of microfluidic chips in a highly controllable and repeatable manner.

### Encapsulation of cells with microgels using the fabricated microfluidic devices

**Fig. 5:**
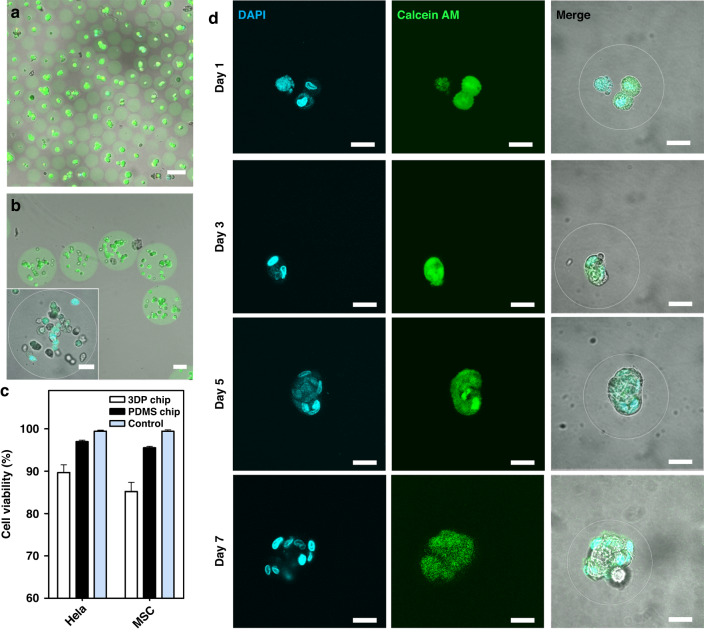
Livability of cells encapsulated in microgels using fabricated microfluidic devices. **a**, **b** Representative confocal microscopic images show MSCs encapsulated in RGD-alginate microgels by 50 μm (**a**) and 300 μm (**b**) DZC-VPP chips, respectively. Scale bars are 100 μm in low-magnification images and 50 μm in high-magnification images. **c** The viability of encapsulated HeLa cells and rat MSCs using DZC-VPP chips versus PDMS chips after encapsulation. **d** Representative confocal microscopic high-magnification images show MSCs encapsulated in RGD-alginate microgels at different time points upon in vitro culture. Scale bars are 20 μm. Calcian-AM stains the MSCs, DAPI stains the cell nucleus, and FITC stains the alginate

We further tested the feasibility of cell encapsulation in fabricated chips with the above-mentioned alginate system. Typically, the chemicals, including monomers, crosslinkers, photoinitiators, and absorbers, can introduce cytotoxicity, especially for the manipulation of live cells, since they might leach out from the chips into the input fluids (Fig. S4, Supporting Information)^43^. To address this problem, we used the fabricated chip to generate cell-laden microgels to evaluate the biocompatibility of chips fabricated by the DZC-VPP approach. By controlling the microchannel dimensions of the drop-maker, we were able to produce microgels of various sizes and mediate the numbers of loaded cells in each microgel (Fig. 5a, b). To assess the biocompatibility of this process, the viability of HeLa cells and rat mesenchymal stem cells (MSCs) encapsulated in the microgels were evaluated by Live/Dead staining. Both cells showed a retained cell viability after encapsulation using the printed chips, with more than 85% of cells remaining viable in the RGD-alginate microgels (Fig. 5c); this was slightly lower than the viability of cells encapsulated using the PDMS chip or the control group of cells cultured on tissue culture plate (Fig. 5c). We further demonstrated that the encapsulated cells continuously proliferated in the microgels during the in vitro culture. Specifically, cells entrapped alginate microgels would gradually proliferate into cell clusters in each individual microgels after seven days of culture (Fig. 5d). These results collectively demonstrated microfluidic devices fabricated via DZC-VPP strategy are biofriendly for manipulating fluids containing live cells, and further applications of this fabrication method can therefore be expanded to other cell-related applications, such as organ-on-a-chip technique.

DLP printing has emerged as a powerful tool for the fabrication of microfluidic chips owing to its superiorities, including user-friendly operative nature, desirable fabrication resolution, and high throughput, which can enable high-precision structure manufacturing ats smaller size than other 3D printing technologies, such as melt-electro writing and fused deposition modeling process. However, limitations remain in suboptimal printing fidelity for channels at sub-100-micron size, cost and labor-consuming, and unreliable mechanical stability^29,39,44^. Herein, we presented a novel DZC-VPP strategy to enable DLP fabrication of microchannels with enhanced resolution and mechanical stability with easy-to-operate commercial 3D printers and resins. In comparison to classical fabrication techniques of microfluidic chips based on soft lithography or hot embossing methods^42,45,46^, the automated production capabilities of the 3DP process can effectively avoid structure errors caused by manual manufacturing and reduce manual labor costs. The batch manufacturing process can also greatly improve the production capacity of microfluidic devices, further reducing the time cost for microfluidic device fabrication. Moreover, compared with the conventional DLP printing process, by calculating the UV penetration into the critical irradiation dose for resin photopolymerization, the DZC-VPP strategy can effectively control the penetration dose while improving the utilization rate of UV irradiation, thus eliminating the limitation on the minimum height of the channel layer. With this printing strategy, the resolution of microstructures will be more determined by the resolution of the 3D printer and the analytical models than the type of 3DP resin^39,40^, which broadened the range of resin selection and printing application. As the printing parameters and feasibility verification of printing equipment can be calculated and completed by mathematical modeling containing pre-established parameters of resin materials, sample structures, and the characteristic of DLP printer, which can significantly reduce the application difficulty of DLP technology. Besides, a more refined structure printing (~20 μm, two times of pixel size) can be achieved with the resolution of commercial 3D printers (at 10 μm × 10 μm per pixel in this study) and regular commercial printing resins, which made the DLP process became more applicable for other delicate applications such as single molecule detection or single-cell screening and analysis^17^. These results demonstrate the feasibility and reliability of the DZC-VPP process, and we envision this high-resolution printing strategy will significantly facilitate the fabrication of 3D-printed microfluidic chips, such as drop-maker, microvalve, microfilter, micromixer, etc.

## Conclusion

In summary, we hereby developed a novel DZC-VPP fabrication strategy to enable the 3D printing of microchannels with enhanced resolution and mechanical stability. Specifically, we proposed an improved mathematical model to precisely predict the accumulated UV irradiance for resin polymerization, which provided guidance for designing the printing process by fine-tuning the dose of UV irradiance and selecting the UV exposure region and depth. This approach allowed to print of microchannel with a cross-section as small as 20 μm × 20 μm just using normal, commercially available 3D printers (10 μm × 10 μm per pixel) and resins and substantially enhanced the scalability of microchannel fabrication compared to that obtained by conventional soft lithography or hot embossing methods. We further demonstrated that the printed chip can be used for the high-throughput generation of monodisperse droplets and cell-laden microgels. This simple but highly efficient microfabrication strategy represents a significant step for the high-resolution and scale-up fabrication of microfluidic devices and could enable advances in the use of microfluidics-based techniques in widespread applications, including organ-on-a-chip, 3D bioprinting, and microencapsulation applications.

## Materials and methods

### Materials

1H,1H,2H,2H-perfluoro-1-octanol (PFO), arginine-glycine-aspartic acid (Arg-Gly-Asp, RGD), sodium alginate, and 4-(2-hydroxyethyl)piperazine-1-ethanesulfonic acid (HEPES) were purchased from Sigma‒Aldrich (USA). Dulbecco’s modified Eagle’s medium (DMEM), minimal essential medium α (α-MEM), fetal bovine serum (FBS), and penicillin/streptomycin were purchased from Gibco (USA). Disodium-EDTA, lysis buffer, 1-ethyl-(dimethylaminopropyl) carbodiimide (EDC), and *N*-hydroxy-sulfosuccinimide (Sulfo-NHS) were purchased from Solarbio (China). Fluorocarbon oil (Novec HFE 7100 Engineered Fluid) was purchased from 3 M (USA). Polydimethylsiloxane (PDMS, RTV-615) was purchased from Momentive (USA). Calcium chloride, ethylenediaminetetraacetic acid disodium salt (Na_2_EDTA), sodium hydroxide, acetic acid, and all the other chemicals were purchased from DAMAO (China).

### 3D printer and resin

A DLP printer (nanoArch S140, wavelength 405 nm, BMF Material Technology Inc., Shenzhen, China) was used to manufacture the microfluidic chips. The minimal theoretical optical lateral resolution of the DLP printer is 10 µm, and the step distance can be tailored in the range of 10–40 µm. The maximum printing vat was 94 mm × 52 mm. Each chip was printed by a series of independent images (1920 × 1080 pixels). All devices were immersed in the resin during the printing process. A commercial transparent resin composed of polymeric urethane acrylate, 2-methyl-1,8-octanediol-diacrylate, diphenyl(2,4,6-trimethylbenzoyl)phosphine oxide, 4-acryloylmorpholine, and tris[2-(acryloyloxy)ethyl]isocyanurate (HTL, BMF Material Technology Inc., Shenzhen, China) was used for the fabrication.

### Measurement of optical characteristic parameters of resin

According to Eq. 1, the characteristic penetration depth of the resin *h*_*a*_ and the critical irradiation dose *D*_*c*_ were calculated based on the polymerization depth of commercial resin at different exposure times and optical irradiance. The optical irradiance was set to 10, 15, 20, 25, and 30 mw/cm^2^, and different exposure times (0.8 to 1.2 s) were applied to fabricate square-shaped single-layer resin samples. Thus, the polymerization depth *z* could be measured under a microscope.

### Tensile tests

The mechanical tests were based on ASTM F1473-18, wherein we also adapted this standard method to our printed samples. Microchannel samples were printed using both conventional DLP and DZC-VPP strategies for the tensile tests. The height of the chips was 5 mm, with the middle part of the chip (thickness of 200 μm, containing a 100 × 100 μm straight channel) printed using different processing strategies. For the conventional DLP method, the printing parameters for the bottom layers were optical irradiance *I* = 20 mw/cm^2^, exposure time *t* = 1.0 s, and layer thickness *z* = 20 μm. The printing parameters in the channel and roof layers were set as optical irradiance *I* = 20 mw/cm^2^, exposure time *t* = 0.5 s, and layer thickness *z* = 20 μm. For the DZC-VPP method, the parameters are introduced in Section 2.2. All mechanical tests were performed by a universal testing machine (E43, MTS instrument, USA) equipped with a 50 N load cell. Fracture energy was used to characterize the work required to break a sample per unit volume, which can be quantified by the area under the tensile stress‒strain curve. The tensile rate was set at a speed of 0.083 1/s.

### 3D printing microfluidic chips

3D models (Fig. S5, Supporting Information) of chips were designed by Autodesk CAD (Autodesk Inc., San Rafael, CA). Slicing software BMF_3D slice (BMF Material Technology Inc., Shenzhen, China) was used to prepare the projection file. The pictures segmented of cover layers were trimmed using Adobe Photoshop CS6. We printed 16 droplet microfluidic chips per batch, which took less than 6 h. The size of each chip was 20 mm × 10 mm (Fig. 4a). The parameters for DZC-VPP printing are introduced in Section “Design rationale and experimental setup for microchannel fabrication using the DZC-VPP strategy”. The diameters of the inlets and outlets were 0.97 mm to allow direct connection to the linker tubes.

### Post-processing of 3D printing microfluidic chips

The uncured resin remaining in the channel was removed by two washing steps with isopropanol after the printing process. The microfluidic chip was subsequently flushed with deionized water to remove IPA, and the remaining solvents were evaporated at 80 °C for 10 min. Sterilization was performed under UV light (365 nm, 100 mW/cm^2^) for 15 min. The morphology of the microfluidic chip was observed by SEM (Nova NanoSEM 450, FEI, USA). To obtain a hydrophobic microchannel surface, a commercial hydrophobic saline Aquapel® (PPG Industries, PA, USA) was injected into the channel (Fig. S6, Supporting Information), followed by incubation at 80 °C for 2 h.

### Fabrication of PDMS microfluidic devices

PDMS devices were fabricated by a soft lithography protocol^18^. Negative photoresist SU-8 (MicroChem, USA) was spin-coated onto a clean silicon wafer to thicknesses of 25, 50, 100, and 300 μm and then UV exposed through a mask (Newway, China) designed by CAD software (Art Service, USA). After developing the microstructure, a 10:1 mixture of polydimethylsiloxane (PDMS, RTV-615, 3 M, USA) and crosslinker were poured onto the pattern and solidified overnight at 85 °C. PDMS molds were peeled off the master, and the channel inlets and outlets were made by using a 1 mm diameter biopsy punch (Wenhan, China). The PDMS replicas were bonded to a glass slide or another PDMS replica after oxygen-plasma activation of both surfaces and cured for one hour at 85 °C. Hydrophobic silane Aquapel (PPG Industries, USA) was injected into the channel and incubated for 90 s at room temperature to render the channel surface hydrophobic. Finally, to totally remove Aquapel, all devices were incubated at 85 °C for ~90 min.

### Generation of droplets and microgels using 3D printing microfluidic chips

For the microfluidic generation of droplets, pure water was used as the aqueous phase, and fluorinated oil HFE7100 consisting of 1 w/v% Krytox-PEG-Krytox surfactant was used as the oil phase^47^. Krytox-PEG-Krytox surfactant was synthesized by the previous^47,48^. For alginate microgels generation, a solution containing 1 w/v% Na-alginate and 50 mM Calcium-EDTA as a crosslinker was used as the aqueous phase, the oil phase is fluorinated oil HFE7100 containing 0.1 v/v% acetic acid and 10 v/v% PFO as surfactant^18^. Another aqueous phase was used for demulsification and separation of microgels from the oil phase. All products were removed to another container for storage. All syringes were connected to the 3D printing devices via polyethylene tubes with an inner diameter of 0.38 mm. The flow rates of aqueous phase *Q*_*aqu*_, oil phase *Q*_*oil*_, and demulsifier phase *Q*_*de*_ were individually controlled by different syringe pumps (lsp-1b, Longer, China). *Q*_*aqu*_ was kept constantly at 100 μL h^−1^, and *Q*_*oil*_ and *Q*_*de*_ are both kept at 1000 μL h^−1^. The high-speed camera and optical microscope were used to monitor the formation and separation of microgels.

### Cell encapsulation in microgels using the printed microfluidic chips

Cell culture experiments were carried out in accordance with the Biomedical and Animal Ethics Committee of Dalian University of Technology (2021-097). In brief, an aqueous phase composed of alginate precursor dissolved in cell culture medium (α-MEM for rat MSCs and DMEM for HeLa cells) was used, which was thereafter dispersed with cells. Specifically, alginate conjugated with a cell attachment motif (arginine-glycine-aspartic acid, RGD) was used for cell culture. During the fabrication process, products were removed to another container for storage. Another buffering aqueous phase containing 20 mM HEPES (pH = 7.4) was added into the container to neutralize the acid and retain the encapsulated cells in the aqueous phase. Cells were mixed into the aqueous phase at a density of 2 × 10^6^ per ml. Cell-laden microgels were finally collected using a cell strainer and then redispersed in cell culture media. The medium was refreshed every three days during in vitro cell culture. Cells in the microgels were cultured for seven days. The viability of the encapsulated cells in alginate microgels was measured by a live/dead assay using a LIVE/DEADs® kit (Invitrogen, USA). The cells were stained with DAPI, and alginate was stained with FITC (Thermo Fisher, USA). Fluorescence images were captured using a confocal laser scanning microscope (OLYMPUS FV1000, Japan).

### Statistical analysis

All data were presented as mean ± standard deviation. Data were analyzed by GraphPad Prism 5. Statistical differences were analyzed with the one-way analysis of variance (ANOVA) and Tukey’s post hoc test. *p* < 0.05 was considered statistically significant.

### Supplementary information

SUPPLEMENTAL MATERIAL
