# Supplementary material for: Digital light processing 3D printing for microfluidic chips with enhanced resolution via dosing- and zoning-controlled vat photopolymerization
Source: Microsyst Nanoeng. 2023 Aug 15;9:103. doi: 10.1038/s41378-023-00542-y (PMC10427687; doi:10.1038/s41378-023-00542-y)
Supplement: Supplementary file 1 — SUPPLEMENTAL MATERIAL [file 41378_2023_542_MOESM1_ESM.docx]

**Supporting Information**

**Digital Light Processing 3D Printing for Microfluidic Chips with Enhanced resolution via Dosing- and Zoning-controlled vat Photopolymerization**

*Zhiming Luo ^1, §^, Haoyue Zhang ^2, §^, Runze Chen^2^, Hanting Li ^2^, Fang Cheng^2^, Lijun Zhang^3^, Jia Liu^4^, Tiantian Kong^1^, Yang Zhang ^1*^, Huanan Wang ^1,2*^*

^1^ *School of Biomedical Engineering, Shenzhen University Health Science Center, Shenzhen 518000, P. R. China*

^2^ *Key State Laboratory of Fine Chemicals, School of Bioengineering, Dalian University of Technology, Dalian 116024, P. R. China*

*^3^ Third People's Hospital of Dalian, Dalian Eye Hospital, Dalian, 116024, P. R. China*

*^4^ Central Laboratory, The Second Affiliated Hospital of The, Chinese University of Hong Kong, Shenzhen 518172, P. R. China*

^∗^ *Author to whom any correspondence should be addressed. Corresponding authors: Yang Zhang (matthew.yang.zhang@gmail.com); Huanan Wang (huananwang@dlut.edu.cn)*

*^§^ These authors contributed equally to this work.*

**Table.1** The corresponding h_a_ and D_c_ values for the resin calculated by Eq.1.

| **Optical irradiance (****I, mW/cm^2^)** | **Characteristic penetration depth (h_a_,** **μm)** | **Critical dose (D_c_, mJ/cm^2^)** |
| --- | --- | --- |
| 10 | 33.63 ± 0.14 | 5.42 ± 0.10 |
| 15 | 34.12 ± 0.10 | 7.72 ± 0.12 |
| 20 | 33.94 ± 0.17 | 8.68 ± 0.12 |
| 25 | 33.79 ± 0.21 | 9.59 ± 0.16 |
| 30 | 34.32 ± 0.22 | 10.23 ± 0.19 |


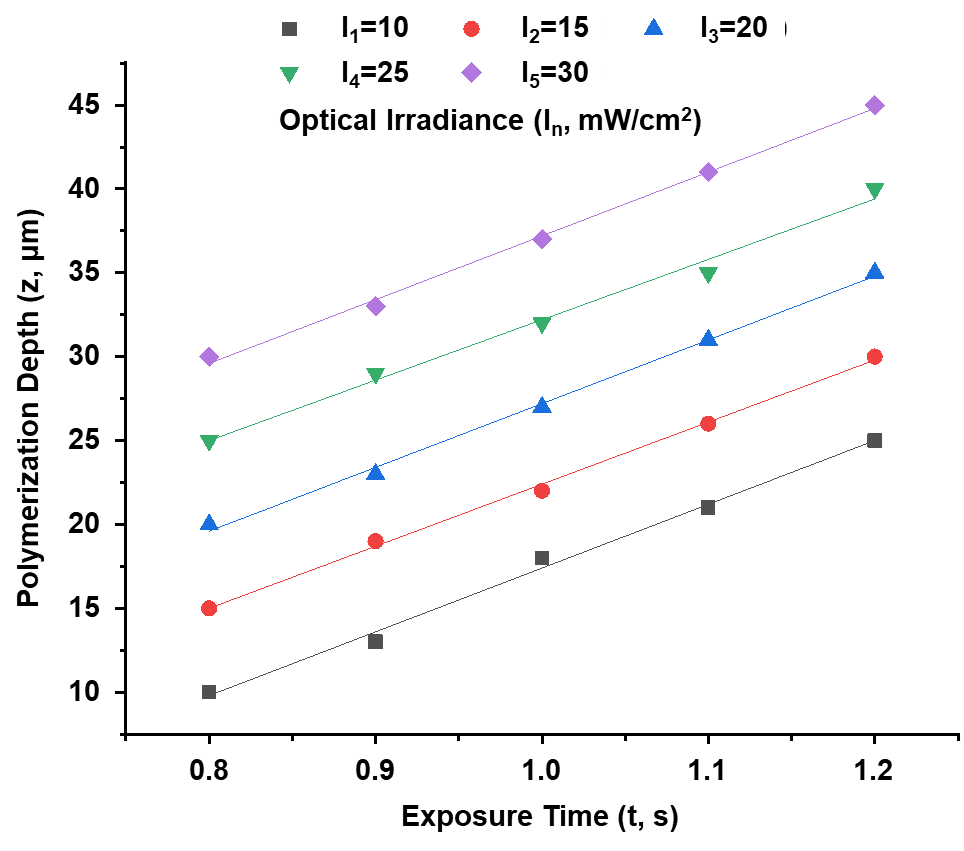


**Figure S1** The relationship between exposure time (t, s) and polymerization depth (z, μm) of resin (HTL, BMF Material Technology Inc) with different optical irradiance (I, 10-30 mW/cm^2^). Under the same optical irradiance (I), the polymerization depth (L) of the resin increases linearly with the exposure time (T). Meanwhile, under the same exposure time (t), the increase of irradiance also leads to the increase of polymerization depth. Therefore, we can calculate and obtain the characteristic penetration depth (h_a_, μm) and the critical dose (D_c_, mJ/cm2) under the different UV optical irradiance by the polymerization depth gathered within this figure, and the results are shown in the Table S1.


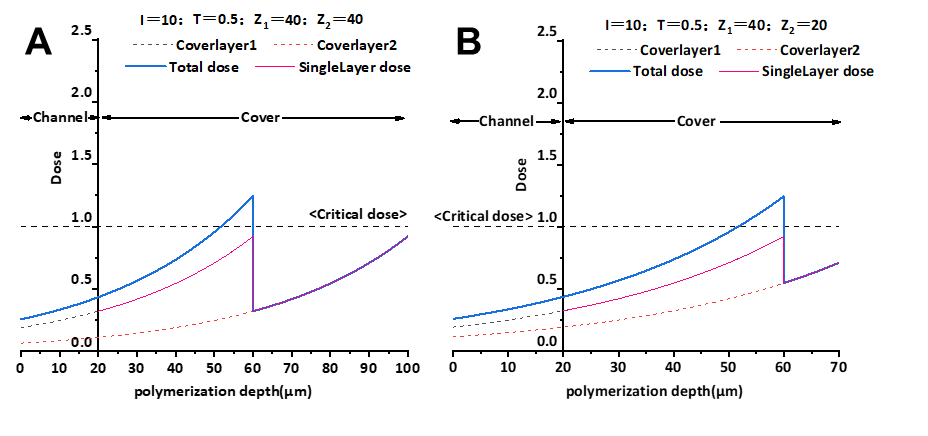


**Figure S2** The distribution of UV irradiance of each projection along the vertical direction that accumulated for the fabrication of the channel-layers and roof-layers using the equal step distance z = 40 μm (A) in comparison with DZC-VPP strategy processing with a connection of step distance z = 20 μm (B). These long step distance process will cause insufficient polymerization of resin and result in a poor strength of the fabricated chip.


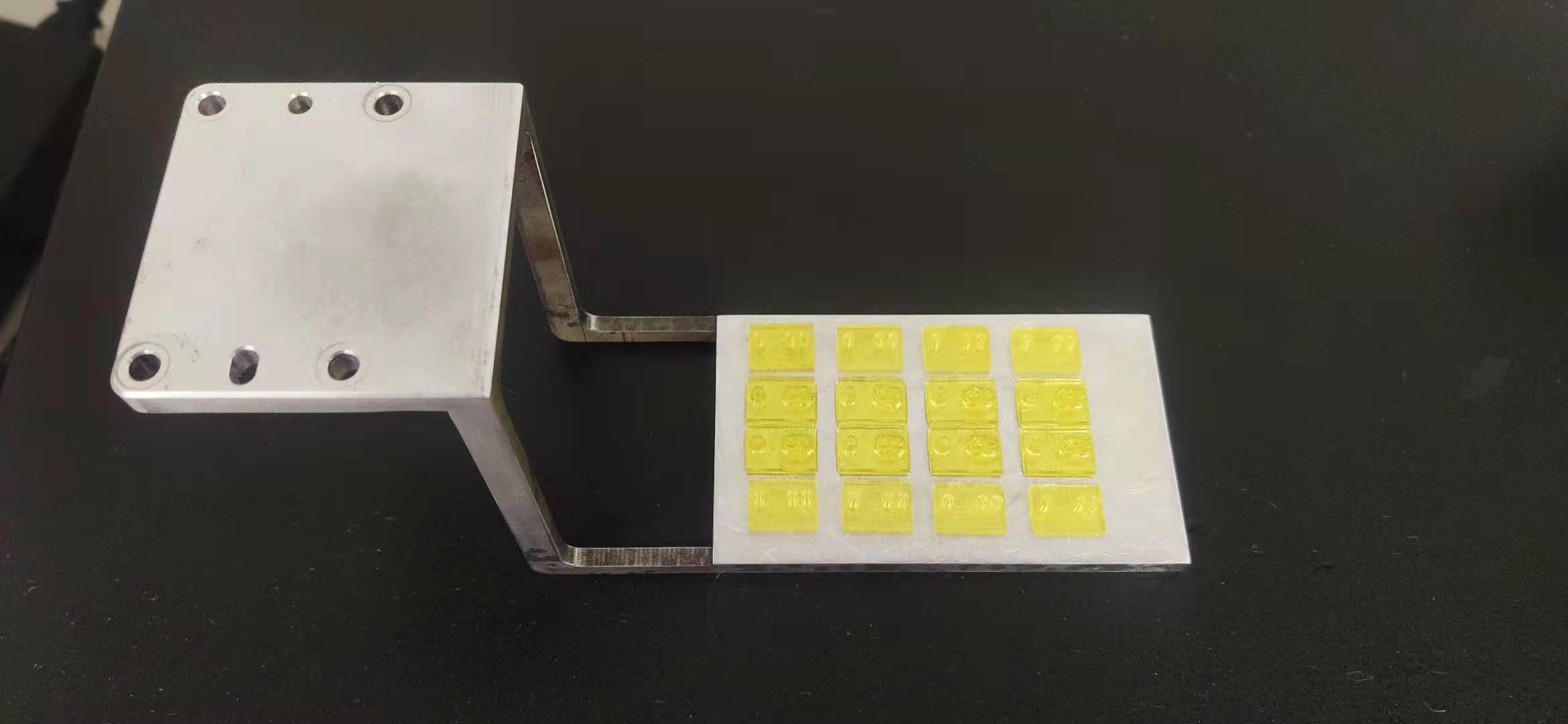


**Figure S3** 16 microfluidic chips with the channel dimension of 20 μm × 20 μm (1 cm × 1.9cm each) were printed within 6 hours, and the defective rate was 0%.


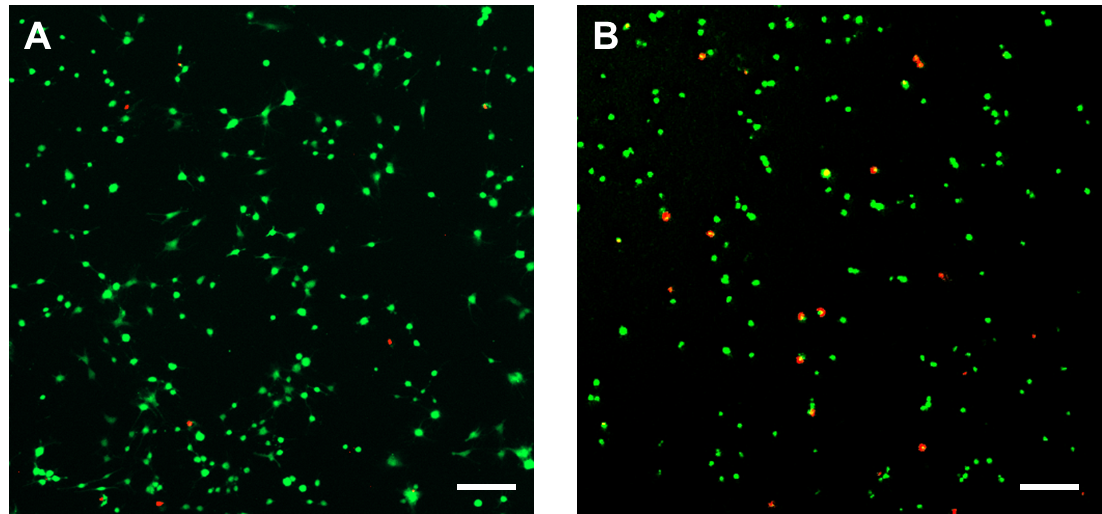


**Figure S4** The viability of rat MSCs cultured in ordinary medium (A) and conditioned medial of DZC-VPP chips after 24 hours (B). The conditioned media is obtained by mixing chips with culture medium and incubated 24h at 37 °C. The cell viability is assessed based on live/dead assay. MSCs cultured by conditioned medial of DZC-VPP chips shows a large range of cell apoptosis, and the proliferation rate was also significantly slower.


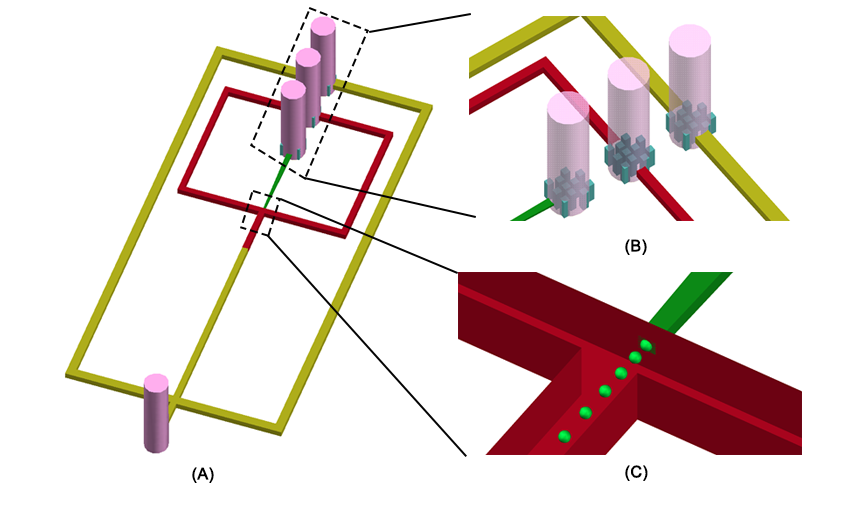


**Figure S5** 3D-schematic of the designed microchannels (A), the filter columns design (B), and the nonplanar flow-focusing junction (C).


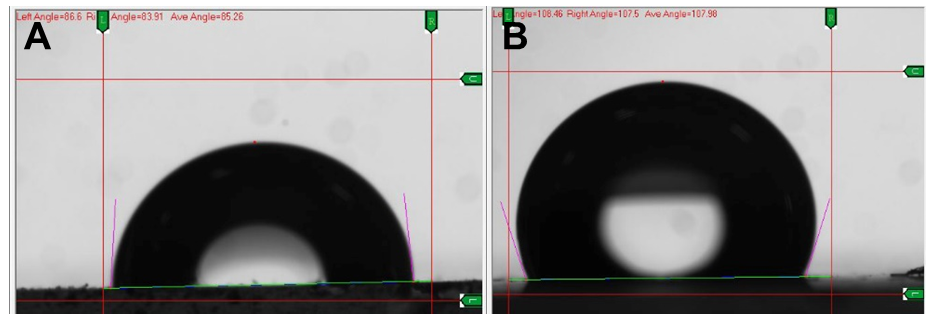


**Figure S6** Contact angle measurement of 3DP chip before (85.26 °, A) and after hydrophobic treatment by Aquapel (107.98°, B).
